# Modified Transseptal Puncture Technique in Challenging Septa: A Randomized Comparison to Conventional Technique

**DOI:** 10.1155/2017/2351925

**Published:** 2017-01-30

**Authors:** Vikas Kataria, Benjamin Berte, Yves Vandekerckhove, Rene Tavernier, Mattias Duytschaever

**Affiliations:** ^1^Department of Cardiology, St Jan Hospital Bruges, Ruddershove 10, 8000 Bruges, Belgium; ^2^University Hospital of Ghent, Ghent, Belgium

## Abstract

*Background*. Transseptal puncture (TSP) can be challenging. We compared safety and efficacy of a modified TSP technique (“mosquito” technique, MOSQ-TSP) to conventional TSP (CONV-TSP).* Method.* Patients undergoing AF ablation in whom first attempt of TSP did not result in left atrial (LA) pressure (failure to cross, FTC) were randomized to MOSQ-TSP (i.e., puncture of the fossa via a wafer-thin inner stylet) or CONV-TSP (i.e., additional punctures at different positions). Primary endpoint was LA access. Secondary endpoints were safety, time, fluoroscopic dose (dose-area product, DAP), and number of additional punctures from FTC to final LA access.* Result.* Of 384 patients, 68 had FTC (MOSQ-TSP, *n* = 34 versus CONV-TSP, *n* = 34). No complications were reported. In MOSQ-TSP, primary endpoint was 100% (versus 73.5%, *p* < 0.002), median time to LA access was 72 s [from 37 to 384 s] (versus 326 s [from 75 s to 1936 s], *p* < 0.002), mean DAP to LA access was 1778 ± 2315 mGy/cm^2^ (versus 9347 ± 10690 mGy/cm^2^, *p* < 0.002), and median number of additional punctures was 2 [1 to 3] (versus 0, *p* < 0.002).* Conclusion.* In AF patients in whom the first attempt of TSP fails, the “mosquito” technique allows* effective, safe, and time sparing LA *access. This approach might facilitate TSP in elastic, aneurysmatic, or fibrosed septa.

## 1. Introduction

Transseptal puncture (TSP) is a conventional approach to access the left atrium (LA). This technique is widely used in catheter ablation of atrial fibrillation (AF), left accessory pathways, or ventricular tachycardia.

Despite increasing use, TSP requires an experienced operator and carries the risk of potentially severe complications [1, 2]. The procedure is especially challenging in patients with elastic or aneurysmal septa but also in fibrosed septa due to prior TSP [3–5]. Presence of such septa not only makes the procedure more difficult but also increases the risk of cardiac perforation as excessive septal tenting (when applying pressure over the septum) may result in sudden uncontrolled forward movement of the needle (harpooning). Use of various imaging modalities (transoesophageal echocardiography and intracardiac echocardiography) or special puncture needles (radiofrequency needle, J-shaped guidewire, etc.) has been shown to reduce such complications [6–10]. However these tools increase the cost and/or complexity of the procedure.

Use of a wafer-thin inner stylet through the conventional transseptal needle to puncture the septum might facilitate TSP. The aim of this study is to compare this modified TSP technique using wafer-thin inner stylet (“mosquito” technique, MOSQ-TSP) with the conventional transseptal puncture technique in patients with challenging septa. To the best of our knowledge, this is the first randomized controlled study comparing these two techniques.

## 2. Methods

### 2.1. Study Population and Transseptal Puncture

Patients undergoing TSP for first or repeat pulmonary venous isolation (PVI) for atrial fibrillation formed the population base for the study. After obtaining vascular access, three sheaths were positioned in the right femoral vein. A decapolar and quadripolar catheters were positioned in the coronary sinus (CS) and at the His position (to mark the aortic root), respectively. Immediately prior to all TSPs an intravenous loading dose of 10,000 IU heparin was administered. TSP was done under fluoroscopy guidance (in right anterior oblique view, RAO 30°, and left anterior oblique view, LAO 50°) and under pressure monitoring from the tip of TSP needle. No routine transoesophageal echocardiography (TOE) was used. All TSPs were done using a Brockenbrough needle (SL0 sheath and BRK-1 TS needle, St Jude Medical Inc., St. Paul, MN, USA). Once the site for TSP (fossa ovalis) was identified (typical “jump” of the needle in LAO 50° and parallel position to the CS catheter in RAO 30°), the septum was punctured using the BRK-1 TS needle. Successful TSP was confirmed by the recording of LA pressure. After LA access, a continuous infusion of heparin was started to maintain an activated clotting time (ACT) above 300 seconds throughout the procedure.

**Figure 1:**
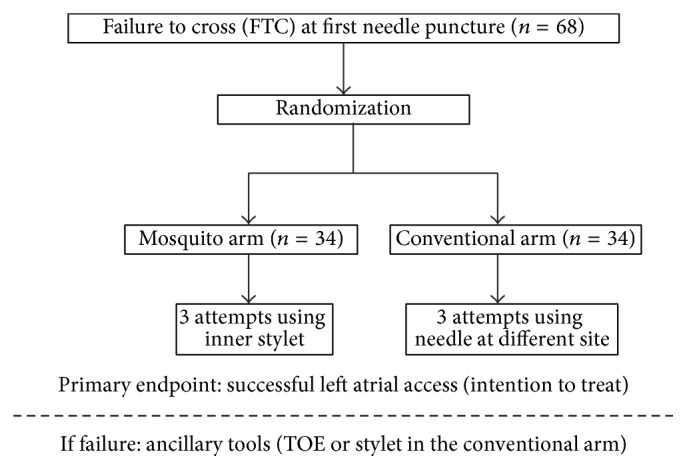
Study flowchart. After failure to cross at first needle puncture, patients were randomized to the mosquito arm or conventional arm. ITT, intention to treat.

Patients with successful TSP at first attempt were not included in the study. Patients in whom first attempt to cross the atrial septum with the conventional needle failed (failure to cross, FTC) formed the study population. At the time of FTC, patients were randomized (by alternation) to undergo TSP either by conventional technique (CONV-TSP group) or by “mosquito” technique (MOSQ-TSP group) (Flowchart, Figure 1). Informed consent was obtained from all patients.

### 2.2. Conventional Technique (CONV-TSP) Group

In the CONV-TSP group, TSP was continued by repeating the same process at different anatomical positions. Success of CONV-TSP was defined if LA access was obtained within a maximum of 3 additional positions. In case of failure of TSP with this technique, puncture was finalised using ancillary tools (TOE or crossover to inner stylet technique). TOE was preferred in case of anesthetized patient whereas crossover to “mosquito” technique was preferred in awake patient.

### 2.3. “Mosquito” Technique (MOSQ-TSP) Groups

**Figure 2:**
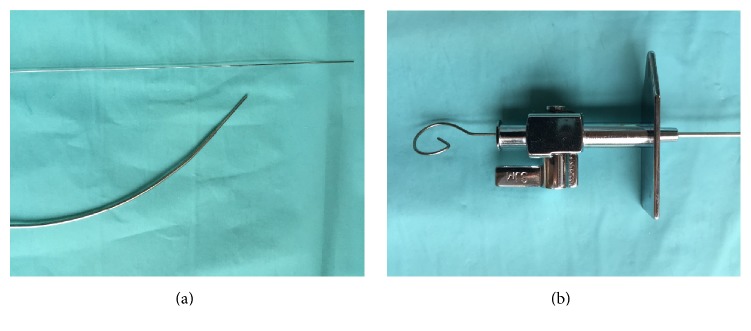
(a) The transseptal needle and its inner stylet. (b) Inner stylet inserted into the needle (to prevent scratching), now used to puncture the already stretched septum.

**Figure 3:**
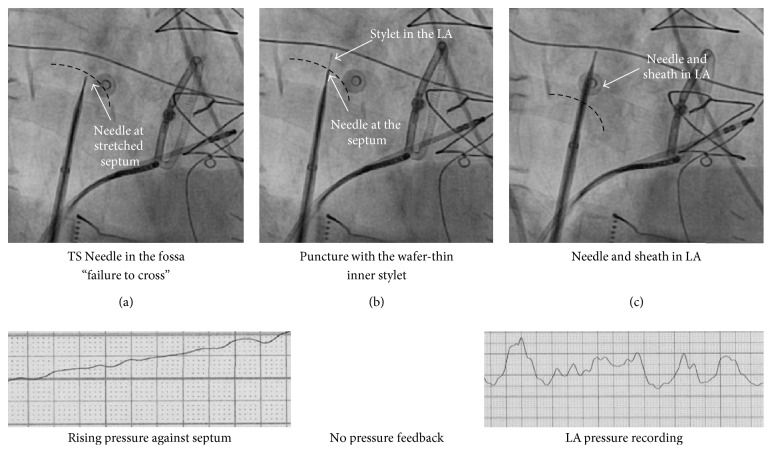
Successful crossing of the septum via the “mosquito” technique. RAO 30° images and pressure recordings from the tip of the needle are presented at various stages of transseptal puncture (from (a) to (c)).

For those patients randomized to MOSQ-TSP, a wafer-thin inner stylet was inserted through the BRK-1 needle at the first position. This inner stylet is the stainless steel stylet delivered together with the BRK™ transseptal needle kit that is used to prevent scratching the inner plastic of the dilator while advancing the needle (Figure 2). The inner stylet was used to puncture the already stretched and tented septum. After manual feedback of crossing the septum, the stylet was withdrawn and the pressure line was reconnected. (Figure 3) Success of MOSQ-TSP was defined as LA access after this single manoeuver. In case of failure of TSP at this first position, puncture could be finalised using ancillary tools (TOE).

### 2.4. Statistical Analysis

In both groups primary endpoint was defined as successful LA access without using ancillary tools (intention to treat analysis). Secondary endpoints were safety, final success rate of TSP (after using ancillary tools), time and fluoroscopic dose from FTC to final LA access (dose-area product, DAP), number of additional needle punctures to final LA access, and need for ancillary tools from FTC to final LA access.

Data are presented as mean ± SD or as percentages or median if data were not normally distributed. Differences between groups were determined by *t*-test and Fisher's exact test. A *p* value of less than 0.05 was considered significant. IBM SPSS version 20.0 was used for statistical analysis.

## 3. Results

### 3.1. Baseline Patient Characteristics

Of 384 patients undergoing TSP for PVI (first procedure, *n* = 320; repeat procedure *n* = 64), 68 patients had FTC at first attempt (first procedure, *n* = 37, 12%; repeat procedure, *n* = 31, 48%). These patients were then randomized to undergo TSP either by conventional method (CONV- TSP group, *n* = 34) or by using wafer-thin inner stylet (MOSQ-TSP group, *n* = 34).

Baseline characteristics of the two groups are shown in Table 1. Mean age (59.7 ± 9.1 versus 59.2 ± 12 years, *p* = 0.82) and gender distribution (males 79.4% versus 73.5%, *p* = 0.60) were similar in both groups. In MOSQ-TSP group 50% had no history of previous TSP (versus 58.8% in CONV-TSP group, *p* = 0.50). In MOSQ-TSP group 41% of procedures were performed under general anesthesia (versus 53% in CONV-TSP group, *p* = 0.16).

### 3.2. Intraprocedural Endpoints (Table 2)

Primary endpoint in the MOSQ-TSP group was 100% (34 out of 34 patients) versus 73.5% in CONV-TSP (25 out of 34 patients, *p* < 0.002). No complications were observed in both groups. Final success rate was 100% in both groups.

Median time from FTC to final LA access was significantly lower in MOSQ-TSP (72 s, range: 37 s–384 s) than CON-TSP (326 s, range: 75 s–1936 s, *p* < 0.002). Similarly, radiation exposure was significantly lower in MOSQ-TSP (mean DAP from FTC to final LA access was 1778 ± 2315 mGy/cm^2^ versus 9347 ± 10690 mGy/cm^2^, *p* < 0.002).

The median number of additional needle punctures from FTC to final LA access was 0 in the MOSQ-TSP group versus 2 in the CON-TSP (range: 1–3, *p* < 0.002).

To achieve LA access in the CONV-TSP group, 11 patients required puncture at one additional site; 20 patients needed puncture at two different sites whereas 3 patients required puncture at 3 extra sites.

In the MOSQ-TSP group, no ancillary tools were required whereas in the CONV-TSP group, initially failed TSP (9 patients, 6 under general anaesthesia, and 3 under local anaesthesia) was finalised using TOE in 6 patients and/or the inner stylet in 6 patients. In 6 patients under general anaesthesia TOE was used. In 3 patients TOE alone was enough to guide TSP, whereas in the other 3 patients TOE + stylet were needed. In 3 patients under local anesthesia, the stylet was used successfully to achieve LA access (crossover).

## 4. Discussion

### 4.1. Main Findings

Our study suggests that the TSP technique using inner stylet (MOSQ-TSP) is a safe, effective, and time sparing method to achieve left atrial access in challenging septa. It can be used as a stand-alone technique without the need of ancillary tools and it markedly reduces the TSP time and radiation exposure.

### 4.2. Challenging Septa

Achieving LA access through transseptal route can be challenging in patients who have an elastic or aneurysmatic septum or a fibrosed septum due to previous procedure. In such conditions, needle puncture stretches the septum without actually crossing it. In some cases, stretching causes the septum to approach close to the lateral LA wall, increasing the risk of perforation [2]. This finding is paralleled in our results: FTC at first attempt occurred in 48% of patients undergoing a repeat procedure, whereas only in 12% of those undergoing TSP for the first time.

Even in expert hands, TSP may be associated with serious and life-threatening complications. Most of the complications are due to inadvertent puncture of structures adjacent to fossa ovalis. These complications may include cardiac tamponade, injury to aorta or atrium, thrombus formation, and air embolism. Most of the case series have reported incidence of serious complications to be 0.5 to 2%. Risk of these complications is higher in atria with distorted anatomy and patients with fibrosed and aneurysmal septa [6]. Risk of complications further increases with every additional attempt of puncture.

Use of ancillary tools to safely cross the challenging septa is common. These tools, however, increase the cost and/or duration of the procedure. Intraprocedural transesophageal echocardiography (TOE) to facilitate TSP requires general anesthesia. This makes the routine use of TOE less feasible in centers where general anesthesia is not used for all patients. Use of intracardiac echocardiography, J-shaped guide wire, or radiofrequency needle has been shown to be safe and effective tools for TSP. [7–10] These tools, however, result in an additional cost burden and increase the procedural time too. Use of electrocautery for TSP has been reported to reduce the failure rate. In a study by Greenstein et al., incidence of tissue coring at septum was found to be as high as 37%. Coring of septal tissue may result in complications such as systemic embolization, raising question about the real safety of this technique [11].

### 4.3. Present Study

The modified TSP technique (mosquito technique) helps to puncture the stretched septum by inserting a wafer-thin stylet through the needle which is already placed at the fossa ovalis. The current study, in our knowledge, is the first one to evaluate the “mosquito technique” in a larger patient group and in a randomized controlled manner. Success rate was 100% at the first puncture site. No TOE was needed, whereas mean time from FTC to LA access could be as long as 32 min in the CONV-TSP group; this time was almost invariably shorter than 3 min in the MOSQ-TSP group. Moreover, total radiation dose was also significantly lower in MOSQ-TSP group. No complications were observed. Absence of perforation can be explained by the fact that the MOSQ-TSP does not require excessive force on the needle, thus avoiding the phenomenon of harpooning after release of the tension following crossing.

This data underlines the fact that in challenging septa MOSQ-TSP is a safe and quick method to achieve successful TSP with significantly less radiation exposure to both patient and the operator. It not only allows the procedure to be done under local anesthesia but also provides significant reduction in procedural time. Moreover, this technique can facilitate TSP in centers where TSP is routinely guided by TOE as well. Finally this approach might be considered as first choice in redo cases especially in centers relying solely on fluoroscopy to cross the interatrial septum.

### 4.4. Limitations

Some centers use TOE in all the patients as the cases are performed under general anesthesia. The benefit of MOSQ-TSP technique, in such cases, may not be of the same magnitude as in the present study.

One could challenge the MOSQ-TSP technique by stating that, using in all patients ICE, J-shaped guidewire, or radiofrequency current (RF) delivery (5–10 W) through the conventional needle, the crossing success rate is 100%. However it is not a matter of being successful in the crossing of challenging septa by this new modality of crossing. Rather, the present study suggests that the MOSQ-TSP technique might save time, fluoroscopy, and cost burden as opposed to other techniques.

## 5. Conclusion

“Mosquito” technique is a modified TS puncture technique allowing safe and rapid LA access in the presence of septal resistance in both primary and repeat ablation. This simplified and costless approach might facilitate TS puncture in the presence of an elastic, fibrosed, or aneurysmal septum.

## Figures and Tables

**Table 1:** Baseline characteristics.

	MOSQ-TSP (*n* = 34)	CONV-TSP (*n* = 34)	*p* value
Age, years	59.7 ± 9.1	59.2 ± 12.0	0.82
Male, *n* (%)	27 (79.4)	25 (73.5)	0.60
Hypertension, *n* (%)	7 (20.6)	9 (26.5)	0.42
Structural heart disease, *n* (%)	8 (23.5)	5 (14.7)	0.32
Diabetes, *n* (%)	0 (0)	2 (5.9)	0.16
CHA_2_DS_2_VASc	1.0 ± 1.2	1.1 ± 1.2	0.89
Atrial diameter (PS-LAX), mm	43.5 ± 6.2	41.7 ± 4.8	0.31
Oral anticoagulants, *n* (%)	22 (64.7)	20 (59)	0.87
1st procedure, *n* (%)	17 (50)	20 (58.8)	0.50
2nd procedure, *n* (%)	13 (38.2)	9 (26.5)	0.40
3rd procedure, *n* (%)	5 (14.7)	7 (20.6)	0.64
General anesthesia, *n* (%)	14 (41.2)	18 (52.9)	0.16
Weight, kg	87.4 ± 18	84.9 ± 17	0.63
TSP for AF ablation, *n* (%)	28 (82.4)	29 (85.3)	0.66

PS-LAX, parasternal long-axis view; AF, atrial fibrillation.

**Table 2:** Procedural outcomes of transseptal puncture.

	MOSQ-TSP (*n* = 34)	CONV-TSP (*n* = 34)	*p* value
Failure to cross at first site, *n* (%)	34 (100)	34 (100)	NS
Successful LA access, *n* (%)	34 (100)	25 (73.5)	<0.002
Final LA access, *n* (%)	34 (100)	34 (100)	NS
Median time from FTC to final LA access, seconds (range)	72 s (37 s–384 s)	326 s (75 s–1936 s)	<0.002
DAP from FTC to final LA access, mGy/cm^2^	1778 ± 2315	9347 ± 10690	<0.002
Median *N* of additional needle punctures	0	2 (1 to 3)	<0.002

FTC, failure to cross the septum at the first attempt of puncture; LA, left atrium; DAP, dose-area product, NS, nonsignificant.
